# Time trends in leisure time physical activity, smoking, alcohol consumption and body mass index in Danish adults with and without COPD

**DOI:** 10.1186/s12890-016-0265-6

**Published:** 2016-07-29

**Authors:** Henrik Hansen, Nina Føns Johnsen, Stig Molsted

**Affiliations:** 1 Research Unit for Chronic Diseases and Telemedicine, University Hospital Bispebjerg and Frederiksberg, Bispebjerg Bakke 23, 2450 Copenhagen, NV Denmark; 2 National Institute of Public Health, University of Southern Denmark, Øster Farimagsgade 5A, 2, Sal 1353 Copenhagen K, Denmark; 3 Department of Clinical Research, Northzealands Hospital, Dyrehavevej 29, 3400 Hillerød, Denmark

**Keywords:** Chronic obstructive pulmonary disease, Physical activity, Time trend, Denmark

## Abstract

**Background:**

Promotion of a healthy lifestyle and non-pharmacological interventions in the treatment of chronic obstructive pulmonary disease (COPD) has received great attention in recent decades. The aim of this study was to investigate trends in leisure time physical activity (PA), smoking, alcohol consumption and body mass index (BMI) from 2000 to 2010 in Danish individuals with and without COPD.

**Methods:**

Analyses were based on data provided by The Danish Health and Morbidity’s three cross-sectional surveys from 2000, 2005 and 2010. Data compromised level of leisure time PA, smoking, alcohol consumption, BMI and sociodemographic characteristics. Participants aged 25 years or older with and without COPD were included in the analyses.

**Results:**

In multiple logistic regression analyses, odds ratio (OR) of being physically active in the leisure time in 2010 compared to 2000 was 1.70 (95 % CI: 1.28-2.26), *p* < 0.001, and 1.32 (1.22-1.43), *p* < 0.001, in participants with and without COPD, respectively. Being a non-smoker in 2010 compared to 2000 was associated with an OR of 1.41 (1.07-1.85), *p* = 0.015, and 1.73 (1.63-1.85), *p* < 0.001, in participants with and without COPD. The OR of not exceeding national recommended alcohol limits was 0.64 (0.45-0.93), *p* = 0.020, and 1.19 (1.09-1.29), *p* < 0.001, in participants with and without COPD. In a multiple linear regression analysis, the time frame from 2000 to 2010 was associated with an increased BMI of 1.18 kg · m^−2^ (0.52-1.84), *p* < 0.001, and 0.74 kg · m^−2^ (0.63-0.86), *p* < 0.001, in participants with and without COPD. The COPD participants with higher levels of education and/or living in a marriage or a relationship were more likely to be physically active, non-smoking and not exceeding the recommended alcohol limits.

**Conclusion:**

From the 2000 to 2010, Danish individuals aged 25 years with and without COPD, increased their leisure time PA level and reduced smoking. Lower socioeconomic status was associated with a reduced level of PA, smoking and an increased alcohol intake. Future national health campaigns and treatment strategies need to target this socioeconomic impact. The reported increased PA level and reduced smoking may have important implications in relation to a reduced morbidity and mortality risk in Danish patients with COPD.

## Background

Physical activity (PA) is a recognized cornerstone in the treatment of patients with chronic obstructive pulmonary disease (COPD) [1–4]. Whilst several studies have reported effects of physical training on physical capacity and quality of life in patients with COPD [3, 5], results from studies on the effect of physical training on PA in patients with COPD are inconsistent [1, 6, 7]. Physical activity has the potential to prevent or delay onset of other chronic diseases, and indeed in patients with COPD, PA is associated with a relatively reduced risk of hospitalization and death [5, 8–11]. The level of daily PA is, however, remarkably reduced in individuals with COPD compared to healthy individuals [12, 13].

Whilst PA is an important element in the treatment of COPD, smoking cessation is crucial in the treatment and prevention of progression of the disease [4]. Furthermore, a relatively high amount of alcohol consumption and to some degree overweight and obesity is associated with an impaired health condition in patients with COPD as in the general population [14, 15]. Indeed physical inactivity, smoking, alcohol consumption and obesity are cardiovascular risk factors and associated with the onset of other chronic diseases including type 2 diabetes, hypertension and dyslipidaemia [10, 15]. Thus, general health campaigns and pulmonary rehabilitation programmes focus on PA, smoking cessation, a limited alcohol intake and a balanced caloric intake.

Whilst the mortality rate has remained unchanged in Danish patients with COPD with approximately 3,500 deaths in 2001 and 3,330 deaths in 2010, the hospital admissions related to COPD were reduced from 22,000 admission in 2001 to 16,000 in 2010 [14, 15]. The reduction in hospitalizations among patients with COPD may be the result of an improved pharmacological treatment, general health campaigns, pulmonary rehabilitation programmes and other non-pharmacologic treatments [1, 4]. It is however not known, whether the patients’ level of PA, percentage of smokers, level of alcohol intake and body weight have changed during the above mentioned time frame.

The primary aim of this study was to investigate whether the level of leisure time PA changed in Danish adults with COPD in the period from 2000 to 2010. In addition, we investigated the trends of smoking, alcohol consumption and body mass index (BMI) during the same time frame. The data from participants with COPD are presented in parallel with data from a Danish population without reported COPD.

## Methods

The data in the present study were provided by The Danish Health and Morbidity Surveys, National Institute of Public Health, University of Southern Denmark [16]. The Danish Health and Morbidity Surveys are nationwide validated surveys conducted every 5 years with approximately 15,000 respondents aged 16 years or older. The survey samples are randomly drawn from the Danish population, registered in the Danish Civil Registration System. In this study, we included data from participants with a minimum age of 25 years from The Danish Health and Morbidity Surveys three cross-sectional analyses from 2000, 2005 and 2010. Data were collected from self-administrated questionnaires and personal interviews in 2000 and 2005 (response rates 74.2 and 66.7 %, respectively), whereas data from 2010 were collected using self-administrated questionnaires (response rate 59.5 %). Written informed consent for participation in the study was obtained from participants before conducting personal interview or self-administrated questionnaires. All data were self-reported and included age, gender, leisure time PA, smoking, alcohol consumption, BMI, level of education, marital status and COPD. Leisure time PA was categorised into four levels: inactive (sedentary leisure activities); moderate active (minimum 4 h of moderate activity weekly as walking, cycling etc.); medium active (minimum 4 h exercise training weekly); and high active (strenuous activity or exercise training several times a week). In order to stratify the participants into being ‘inactive’ or ‘active’ , the three highest levels of leisure time PA (‘moderate’ , ‘medium’ and ‘high active’) were combined into one level [17]. Smoking status was categorised as being current smoker or not. Alcohol consumption was evaluated using Danish national maximal recommendations and categorized as exceeding alcohol limits or not, respectively 7 alcoholic drinks within the past week for females, and 14 drinks for males. Level of education was categorised as: <10 years (~basic school); 10–12 years (~high school); and +13 years (~university). Body mass index (kg^.^m^−2^) was used to estimate body weight. Marital status was categorised and analysed as: “living alone”, “living with partner or being married”. Type of COPD (emphysema, chronic bronchitis and bronchiestasis), COPD severity (GOLD I, II, III, IIII) and classification (Group A, B, C and D) were not reported and data were analysed as participants with “COPD” and “non-COPD”. The study was in accordance with the approval of the local ethical committee and the Danish Data Protection Agency.

### Statistic

Statistical analyses were carried out using PASW® IBM SPSS Statistics 22. Unadjusted time changes in continuous data were analysed using one-way ANOVA, and in categorical data a Chi [2] test were used. Unadjusted differences between participants with and without COPD were analysed using Student’s *t*-test and Chi [2] test. Multiple regression analyses were conducted to control for confounders in time changes in participants with and without COPD. In the regression analyses year 2000 was used as reference of the time changes 2000–2005 and 2000–2010, whereas 2005 was reference in the analyses of 2005–2010. Independent variables included in the time period was age, gender, living with a partner or not, BMI, level of education. As BMI have different reference intervals in the two samples of participants, separated regression analyses were conducted in COPD and non-COPD participants. Estimates of changes in the categorical variables leisure time PA, smoking and alcohol consumption were tested using logistic regression analyses, and effects on the continuous variable BMI were tested using a linear model. All models were tested with and without two-way interactions between the time changes and the other independent variables to investigate whether the associations had interactions between time changes and e.g. gender or age. As there were no significant differences between models with and without interactions, these were not included in the results. Data are presented as the numbers and percentages, the mean ± standard deviation (SD), odds ratio (OR) and confidence interval (CI), or effect size and CI. All tests were two-tailed at a significance level of *p* ≤ 0.05.

## Results

The characteristics of the participants are presented in Table 1. The prevalence of COPD in the studied populations was 3.3 % in 2000, 3.7 % in 2005 and 5.2 % in 2010. The COPD participants were older compared to the non-COPD participants in the three analysed periods. Both groups consisted fewer males than females. The level of education and percentages of subjects, who were married or living with a partner increased from 2000 to 2010 in COPD as in non-COPD participants. The level of education and percentages of subjects who were married or living with partner were consistently lower in participants with COPD compared to the non-COPD participants.

**Table 1:** Characteristics of participants with and without COPD in the years 2000, 2005, and 2010

Variable	2000	2005	2010	*p*
Participants (*n* (%))				
COPD females	265 (55.3)	289 (59.2)	312 (47.9)	
COPD males	214 (44.7)	199 (40.8)	339 (52.1)	
Non-COPD females	7141 (51.1)	6500 (51.4)	6385 (53.5)	
Non-COPD males	6840 (48.9)	6148 (48.6)	5556 (46.5)	
Age (years)				
COPD females	59.5 ± 15.1***	61.6 ± 12.8***	63.3 ± 12.9***	0.004
COPD males	62.8 ± 15.3***	64.6 ± 12.2***	64.3 ± 12.8***	0.296
Non-COPD females	50.4 ± 16.7	51.7 ± 16.4	51.9 ± 15.2	<0.001
Non-COPD males	49.4 ± 15.6	50.6 ± 15.4	52.7 ± 14.8	<0.001
Marital status (*n* (%))				
COPD females				0.207
Married/living with partner	140 (53.2)***	159 (55.2)***	184 (60.3)***	
Living alone	123 (46.8)***	129 (44.8)***	121 (39.7)***	
COPD males				0.039
Married/living with partner	139 (65.0)***	152 (76.4)	234 (70.5)***	
Living alone	75 (35.0)***	47 (23.6)	98 (29.5)***	
Non-COPD females				<0.001
Married/living with partner	5101 (71.8)	4699 (72.3)	4689 (75.1)	
Living alone	2004 (28.2)	1801 (27.7)	1551 (24.9)	
Non-COPD males				<0.001
Married/living with partner	5260 (77.1)	4828 (78.2)	4466 (81.3)	
Living alone	1560 (22.9)	1316 (21.4)	1029 (18.7)	
Educational level (*n* (%))				
COPD females				<0.001
< 10 years	133 (50.6)***	145 (50.9)***	113 (37.9)***	
10-12 years	117 (44.5)***	117 (41.1)***	135 (45.3)***	
13+ years	13 (4.9)***	23 (16.8)***	50 (16.8)***	
COPD males				<0.001
< 10 years	88 (41.7)***	52 (26.4)***	91 (27.5)***	
10-12 years	105 (49.8)***	117 (59.4)***	177 (53.5)***	
13+ years	18 (8.5)***	28 (14.2)***	63 (19.0)***	
Non-COPD females				<0.001
< 10 years	1944 (27.5)	1442 (22.5)	906 (14.6)	
10-12 years	3664 (51.8)	3259 (50.8)	3012 (48.5)	
13+ years	1464 (20.7)	1709 (26.7)	2293 (36.9)	
Non-COPD males				<0.001
< 10 years	1405 (20.8)	982 (16.2)	676 (12.4)	
10-12 years	3931 (58.1)	3612 (59.8)	2999 (55.1)	
13+ years	1431 (21.1)	1450 (24.0)	1767 (32.5)	
Leisure time physical activity (*n* (%))				
COPD females				0.002
Moderate/highly active	161 (62.2)***	206 (72.3)***	231 (75.5)***	
Inactive	98 (37.8)***	79 (27.7)***	75 (24.5)***	
COPD males				0.002
Moderate/highly active	129 (60.8)***	147 (76.6)***	241 (71.7)***	
Inactive	83 (39.2)***	45 (23.4)***	95 (28.3)***	
Non-COPD females				<0.001
Moderate/highly active	5876 (83.2)	5639 (87.5)	5610 (88.6)	
Inactive	1190 (16.8)	809 (12.5)	724 (11.4)	
Non-COPD males				<0.001
Moderate/highly active	5719 (84.5)	5285 (87.0)	4900 (88.6)	
Inactive	1051 (15.5)	793 (13.0)	630 (11.4)	
Daily smokers (*n* (%))				
COPD females	154 (58.1)***	154 (53.5)***	127 (41.5)***	<0.001
COPD males	123 (57.5)***	89 (44.9)***	161 (48.1)***	0.026
Non-COPD females	2260 (31.6)	1752 (26.9)	1152 (18.5)	<0.001
Non-COPD males	2480 (36.3)	1944 (31.6)	1115 (20.3)	<0.001
Exceeding alcohol limits (*n* (%))				
COPD females	24 (9.1)	32 (11.1)	33 (11.8)***	0.576
COPD males	37 (17.3)	49 (24.7)**	77 (23.8)***	0.123
Non-COPD females	576 (8.1)	708 (10.9)	389 (6.3)	<0.001
Non-COPD males	936 (13.7)	1057 (17.2)	672 (12.4)	<0.001
Body mass index (kg · m^−2^)				
COPD females	24.7 ± 5.1	24.8 ± 5.8	25.7 ± 5.6*	0.056
COPD males	25.7 ± 4.4	26.9 ± 5.1*	26.9 ± 4.9	0.007
Non-COPD females	24.3 ± 4.3	24.7 ± 4.5	25.0 ± 4.9	<0.001
Non-COPD males	25.7 ± 3.6	26.0 ± 3.8	26.4 ± 4.2	<0.001
Obesity, BMI ≥30 kg · m^−2^ (*n* (%))				
COPD females	43 (16.6)***	41 (14.3)	56 (18.8)**	0.342
COPD males	28 (13.2)	42 (21.4)***	69 (20.7)**	0.050
Non-COPD females	681 (9.8)	742 (11.5)	827 (13.3)	<0.001
Non-COPD males	708 (10.4)	754 (12.3)	795 (14.5)	<0.001

Data are presented as numbers (percentages) or the mean ± SD. Time changes in categorical data were compared using Chi [2] and time changes in continues data were compared using one-way ANOVA. Participants with COPD significantly different from participants without COPD tested using a Student’s *t*-test for continuous data, and Chi [2] test for categorical data, *p < 0.05, **p < 0.01, and ***p < 0.001

### Change in leisure time physical activity

The percentages of participants, who were physically active in their leisure time increased significantly from 2000 to 2010 in the COPD and in the non-COPD populations (Table 1). The COPD participants were less engaged in leisure time PA compared with the non-COPD participants from 2000 to 2010.

In the adjusted regression analyses an increased level of PA was found to be associated with the observed period from 2000 to 2010 in COPD and in non-COPD populations (OR 1.70 (95 % CI: 1.28-2.26) and OR 1.32 (95 % CI: 1.22-1.43), respectively). Whilst the period from 2000 to 2005 was associated with an increased level of PA, 2005 to 2010 was not associated with a change in the level of PA.

Being physically active was negatively associated with smoking, increasing age and elevated BMI in both COPD and non-COPD participants. An elevated alcohol consumption was negatively associated with PA in the COPD participants (Table 2). In all participants, being married or living with a partner, and elevated level of education was associated with being physically active. Gender was not associated with leisure time PA in the adjusted analysis (Table 2).

**Table 2:** Estimates of being physically active during the leisure time in Danish adults with and without COPD

	COPD			Non-COPD		
Variable	OR	CI 95 %	*p*	OR	CI 95 %	*P*
Time trends (years)						
2000–2005	1.72	1.29; 2.29	<0.001	1.28	1.19; 1.37	<0.001
2005–2010	0.99	0.74; 1.33	0.961	1.04	0.95; 1.13	0.399
2000–2010	1.70	1.28; 2.26	<0.001	1.32	1.22; 1.43	<0.001
Age (years)	0.98	0.97; 0.99	<0.001	0.98	0.98; 0.99	<0.001
Body mass index (kg · m^−2^)	0.96	0.94; 0.98	<0.001	0.95	0.94; 0.95	<0.001
Current smoker	0.64	0.52; 0.86	0.002	0.57	0.53; 0.61	<0.001
Exceeding alcohol limits	0.75	0.54; 1.02	0.069	0.87	0.79; 0.95	0.003
Married/living with a partner	1.45	1.14; 1.85	0.002	1.65	1.54; 1.77	<0.001
Gender (male)	0.97	0.76; 1.24	0.801	1.03	0.97; 1.09	0.364
Education (years)						
< 10 vs. 10-12	0.68	0.53; 0.87	0.002	0.64	0.60; 0.69	<0.001
< 10 vs. +13	0.44	0.29; 0.68	<0.001	0.49	0.44; 0.54	<0.001
10-12 vs. +13	0.65	0.43; 0.99	0.048	0.76	0.69; 0.83	<0.001

Odds of being physically active. Multiple logistic regression analysis

### Change in smoking habits

From 2000 to 2010 the percentages of smokers decreased in the COPD and the non-COPD participants (Table 1). The COPD participants comprised relatively more current smokers compared with the non-COPD participants throughout the observed period.

The estimates of being a non-smoker are presented in Table 3. There were associations between not smoking currently and the period from 2000 to 2010 in the COPD participants (OR 1.41 (95 % CI: 1.07-1.85)) and in the non-COPD participants (OR 1.73 (95 % CI: 1.63-1.85)). Being a non-smoker was found to be associated with increasing age, elevated BMI, being married or living with a partner and an elevated level of education, whereas non-smoking was negatively associated with elevated alcohol consumption, physical inactivity and being male in COPD and non-COPD participants (Table 3).

**Table 3:** Estimates of being a non-smoker in Danish adults with and without COPD

	COPD			Non-COPD		
Variable	OR	CI 95 %	*p*	OR	CI 95 %	*p*
Time trends (years)						
2000–2005	1.22	0.92; 1.61	0.163	1.17	1.11; 1.23	<0.001
2005–2010	1.15	0.88; 1.50	0.294	1.48	1.39; 1.58	<0.001
2000–2010	1.41	1.07; 1.85	<0.015	1.73	1.63; 1.85	<0.001
Age (years)	1.05	1.04; 1.05	<0.001	1.01	1.01; 1.02	<0.001
Body mass index (kg · m^−2^)	1.08	1.05; 1.10	<0.001	1.06	1.06; 1.07	<0.001
Exceeding alcohol limits	0.49	0.36; 0.67	<0.001	0.51	0.47; 0.54	<0.001
Physical activity	0.68	0.53; 0.88	0.003	0.58	0.54; 0.62	<0.001
Married/living with a partner	1.58	1.25; 2.00	<0.001	1.45	1.37; 1.53	<0.001
Gender (male)	0.76	0.60; 0.96	0.021	0.78	0.74; 0.82	<0.001
Education (years)						
< 10 vs. 10-12	0.72	0.14; 1.31	0.015	0.72	0.59; 0.84	<0.001
< 10 vs. +13	0.99	0.09; 1.88	0.030	1.63	1.49; 1.77	<0.001
10-12 vs. +13	0.27	−0.58; 1.11	0.536	0.92	0.81; 1.02	<0.001

Odds for not smoking currently. Multiple logistic regression analysis

### Change in alcohol consumption

The percentages of subjects, who exceeded the recommended alcohol limit, were unchanged from 2000 to 2010 in the participants with COPD, whereas a significant peak in 2005 was observed in the non-COPD participants (Table 1).

In the adjusted analyses, not exceeding the alcohol limits was negatively associated with the period from 2000 to 2010 in COPD populations (OR 0.64 (95 % CI: 0.45-0.93)), whereas a reverse association was found in the non-COPD populations in from 2000 to 2010 (OR 1.19 (95 % CI: 1.09-1.29).

Being current smoker, being male and a higher level of education were negatively associated with not exceeding the alcohol limit in all participants. In addition, physical inactivity was negatively associated with not exceeding the alcohol limit in the non-COPD participants (Table 4).

**Table 4:** Estimates of not exceeding the Danish recommended limitations of alcohol intake in Danish adults with and without COPD

	COPD			Non-COPD		
Variable	OR	CI 95 %	*p*	OR	CI 95 %	*p*
Time trends (years)						
2000–2005	0.62	0.42; 0.91	0.014	0.75	0.69; 0.81	<0.001
2005–2010	1.04	0.74; 1.45	0.834	1.58	1.45; 1.72	<0.001
2000–2010	0.64	0.45; 0.93	0.020	1.19	1.09; 1.29	<0.001
Age (years)	1.01	1.00; 1.02	0.031	0.99	0.99; 0.99	<0.001
Body mass index (kg · m^−2^)	0.99	0.97; 1.03	0.914	0.99	0.99; 1.01	0.849
Current smoker	0.49	0.36; 0.67	<0.001	0.51	0.48; 0.55	<0.001
Physical activity	0.76	0.56; 1.05	0.095	0.89	0.82; 0.99	0.023
Married/Partner	0.96	0.71; 1.30	0.777	1.11	1.03; 1.20	0.006
Gender (male)	0.45	0.33; 0.61	<0.001	0.57	0.53; 0.61	<0.001
Education (years)						
< 10 vs. 10-12	1.23	0.88; 1.70	0.223	1.63	1.48; 1.79	<0.001
< 10 vs. +13	2.25	1.45; 3.48	<0.001	1.82	1.63; 2.03	<0.001
10-12 vs. +13	1.83	1.23; 2.73	0.003	1.12	1.04;1.21	0.004

Odds for not exceeding alcohol limits. Multiple logistic regression analysis

### Change in body mass index

During the period from 2000 to 2010 BMI increased in the unadjusted analyses in all participants with the exception of females with COPD. The mean BMI were comparable between the COPD and non-COPD participants (Table 1).

The results from the adjusted analysis on the effects on BMI are presented in Table 5. The period from 2000 to 2010 was associated with an increased BMI in the COPD and the non-COPD participants, respectively 1.18 kg · m^−2^ (95 % CI: 0.52-1.84) and 0.74 kg · m^−2^ (95 % CI: 0.63-0.86). In all participants, physical inactivity and being male was associated with an increase in BMI, whereas smoking was associated with a reduced BMI.

**Table 5:** Effects on body mass index in Danish adults with and without COPD

	COPD			Non-COPD		
Variable	Effect	CI 95 %	*p*	Effect	CI 95 %	*p*
Time trends (years)						
2000–2005	0.56	−0.11; 1.23	0.102	0.35	0.25; 0.46	<0.001
2005–2010	0.62	−0.22; 1.26	0.059	0.39	0.29; 0.49	<0.001
2000–2010	1.18	0.52; 1.84	<0.001	0.74	0.63; 0.86	<0.001
Age (years)	−0.06	−0.08; −0.04	<0.001	0.01	0.004; 0.01	<0.001
Current smoker	−1.87	−2.42; −1.32	<0.001	−0.98	−1.08; −0.88	<0.001
Exceeding alcohol limits	0.04	−0.69; 0.77	0.919	0.00	−0.13; 0.14	0.969
Physical activity	0.93	0.34;1.53	0.002	0.98	0.85;1.11	<0.001
Married/living with a partner	0.13	−0.44; 0.69	0.661	0.34	0,23; 0.44	<0.001
Gender (male)	1.68	1.13; 2.23	<0.001	1.38	1.29; 1.47	<0.001
Education (years)						
< 10 vs. 10-12	0.72	0.14; 1.31	0.015	0.72	0.60; 0.84	<0.001
< 10 vs. +13	0.99	0.09; 1.88	0.030	1.63	1.49; 1.77	<0.001
10-12 vs. +13	0.27	−0.58; 1.11	0.536	0.92	0.81; 1.02	<0.001

Effects on BMI. Multiple linear regression analysis

## Discussion

The present study on participants with COPD suggests that the time frame from 2000 to 2010 was associated with greater odds of being physically active and being a non-smoker. Furthermore, the period from 2000 to 2010 was found to be associated with greater odds of exceeding the Danish recommended maximum alcohol intake and an elevated BMI among participants with COPD.

To our knowledge this is the first study to present an increased level of PA over a decade in national samples of participants with COPD. Similar findings of increases in PA in general population surveys have been reported in studies from Denmark, Sweden, Finland, England and Canada in recent decades [18–25]. Individuals with COPD are significantly less physically active compared to healthy sex and age matched individuals, even in the mild stages of COPD [12, 13, 26]. Our study confirms this assumption, since a markedly greater percentage of the non-COPD participants reported to be moderate or highly active compared to the COPD samples. Thus, Danish persons with COPD remain less a physically active than those without COPD, and there are still a need to focus on this aspect in national and local treatment strategies. However, the trend of an elevated level of PA over the covered decade was found to be more pronounced in COPD vs. non-COPD samples when the OR’s are compared. Indeed the underlying reason to the presented increased leisure time PA is difficult to point out. We have two qualifying suggestions to contributing factors to the increased level of PA: First, our study finding may be the result of a national health campaign promotion of “30 min of PA per day” which began around the millennium in Denmark. Second, at the same point of time an increased effort in rehabilitation including PA to patients with chronic diseases treated in hospitals, municipalities and in general practitioners was prioritized politically. Indeed the above mentioned general and chronic disease specific focus on PA may be associated with an elevated level of PA in individuals with COPD.

Another finding in our study suggests a socioeconomic impact on the participants’ reported level of PA. The COPD participants with higher education and/or a partner in a marriage or a relationship were more likely to be physically active. Furthermore, unhealthy lifestyle including smoking, exceeding alcohol limits and elevated BMI reduced the likelihood of being physically active. This could indicate that future national health campaigns that promote PA should target particularly lower socioeconomic population groups with i.e. COPD.

The time frame was found to be associated with reduced smoking in COPD and non-COPD samples. One such positive trend may be the result of decades’ information campaigns to the general population in a combination with recent year politics, which have forbidden smoking in a long list of public and private areas. Reduced smoking among subjects with COPD may not only affect the mortality rate positively in terms of a reduced risk, but reduced smoking may also prevent progressions of the COPD and reduce disease progressions and specific complications. Whilst the time trend in smoking was the same in samples with or without COPD, the time period from 2000 to 2010 was associated with elevated odds of exceeding the recommended maximum alcohol intake in samples with COPD, whereas the odds were reduced in non-COPD participants. We hypothesize that these different associations may be related to the participants’ level of education. Recent Danish general health campaigns have not only focused on the benefits of PA and smoking cessation but also on a reduced alcohol intake. However, traditional health campaigns may have the most pronounced effect in individuals with a higher level of education. Thus, as a result of the reduced educational level in participants with COPD compared to those without COPD, a reduction in the alcohol intake may primarily be found in the higher educated non-COPD samples in the covered decade. The association between the time period from 2000 to 2010 and the elevated BMI was found in COPD and in non-COPD participants. It is likely that an elevated BMI is preferable in underweight individuals with COPD. However, in this study ~20 % of the participants with COPD were in 2010 found to be obese, a finding that needs attention in clinical practice as obesity is associated with other chronic diseases including type 2 diabetes, hypertension and dyslipidemia.

The present positive time trend in elevated level of PA and reduced smoking in individuals with COPD may have important clinical implications. An increased level of PA and reduced smoking are associated with reduced morbidity and mortality [4, 10, 11, 27–30]. Thus, if the mortality rate in Danish COPD samples will decrease after the 2010 it may be associated with elevated level of PA and reduced smoking. On the other hand, an impact on the mortality rate may also be associated negatively with the reported greater alcohol intake and obesity. Another positive effect of the increase in PA could be an improvement in physical function. Many patients with COPD may have a reduced physical function related to daily activities as a result of reduced PA and reduced muscle strength. The patients’ muscle strength may be reduced by muscle atrophy caused by elevated levels of TNF-α and other cytokines and physical inactivity. The importance of the prevention of loss of physical functions is very important now and in the future with the increasing number of older persons all over the world.

The present study had several strengths. The data were collected from a nationally representative survey to monitor status and development in health related behavior in Denmark, and the COPD prevalence was stable over time and corresponds to national reported data ranging from 4–6 % in the analyzed time period [14]. Furthermore, the observed changes covered a period of a whole decade. On the other hand, the study has some important methodological limitations due to the nature of the protocol. The results may be biased due to the absence of data from non-responders and a reduced response rate in 2010 compared to the two earlier cohorts, which may have resulted in analyses of selected samples without being representative. Another important limitation in the study is that COPD severity not was reported by the participants. Finally, self-reported data used as a measure of change in PA, smoking, alcohol habits and BMI do not have the same validity as data obtained during directly measurements or observations. However, this is often not feasible in nationwide representative surveys due to extensive cost.

## Conclusion

In conclusion, this study found that the time frame from 2000 to 2010 was associated with an elevated level of leisure time PA and reduced smoking in Danish participants with and without COPD included in three nationwide surveys. The positive trends in self-reported PA and smoking habits may have important clinical implications in terms of reduced morbidity and mortality. Whilst the time trend was associated with more participants with COPD who exceeded the recommended national maximum of alcohol intake, the time trend was associated with a reduced alcohol intake in participants without COPD. The positive trend in elevated level of PA and reduced smoking may be the result of national general health campaigns and COPD specific rehabilitation programs.

## Abbreviations

BMI, body mass index; CI, confidence interval; COPD, chronic obstructive pulmonary disease; OR, odds ratio; PA, physical activity; SD, standard deviation
